# Intranasal Vaccination Strategy to Control the COVID-19 Pandemic from a Veterinary Medicine Perspective

**DOI:** 10.3390/ani11071876

**Published:** 2021-06-24

**Authors:** Salleh Annas, Mohd Zamri-Saad

**Affiliations:** Department of Veterinary Laboratory Diagnosis, Faculty of Veterinary Medicine, Universiti Putra Malaysia, Serdang 43400, Selangor, Malaysia; mzamri@upm.edu.my

**Keywords:** intranasal, vaccine, pandemic, COVID-19, coronavirus

## Abstract

**Simple Summary:**

Intranasal vaccination is one of the methods used to stimulate mucosal immunity. It has been widely practised to control many human and animal respiratory diseases. Coronavirus disease 2019 (COVID-19) is a highly contagious respiratory disease caused by severe acute respiratory syndrome coronavirus 2 (SARS-CoV-2), which resulted in a global pandemic. COVID-19 has reminded some veterinarians of various contagious veterinary diseases, including coronavirus infections in animals. This article discusses the control of highly contagious diseases of veterinary importance with emphasis on an intranasal vaccination approach, and the potential of implementing similar strategies in human medicine to control the ongoing COVID-19 pandemic.

**Abstract:**

The world is currently facing an ongoing coronavirus disease 2019 (COVID-19) pandemic. The disease is a highly contagious respiratory disease which is caused by severe acute respiratory syndrome coronavirus 2 (SARS-CoV-2). Current control measures used by many countries include social distancing, wearing face masks, frequent hand washing, self-isolation, and vaccination. The current commercially available vaccines are injectable vaccines, although a few intranasal vaccines are in trial stages. The reported side effects of COVID-19 vaccines, perceptions towards the safety of the vaccines, and frequent mutation of the virus may lead to poor herd immunity. In veterinary medicine, attaining herd immunity is one of the main considerations in disease control, and herd immunity depends on the use of efficacious vaccines and the vaccination coverage in a population. Hence, many aerosol or intranasal vaccines have been developed to control veterinary respiratory diseases such as Newcastle disease, rinderpest, infectious bronchitis, and haemorrhagic septicaemia. Different vaccine technologies could be employed to improve vaccination coverage, including the usage of an intranasal live recombinant vaccine or live mutant vaccine. This paper discusses the potential use of intranasal vaccination strategies against human COVID-19, based on a veterinary intranasal vaccine strategy.

## 1. Introduction

The current coronavirus disease 2019 (COVID-19) pandemic is caused by severe acute respiratory syndrome coronavirus 2 (SARS-CoV-2). The disease started in late 2019 in Wuhan, China [1], and a pandemic was later declared by the World Health Organization in March 2020 [2]. The disease is basically a respiratory disease, which is rapidly transmitted by oral and nasal droplets as the infected person breathes, coughs, sneezes, or speaks, and the common portals of entry include the mouth, nose, or eyes [3]. Once these facts were established, immediate recommendations such as preventive measures including social distancing, the wearing of face masks, frequent hand washing, surface disinfection, and the self-isolation of persons exhibiting clinical signs associated with the disease were implemented [4]. These are regarded as temporary preventive measures due to the need to co-exist with SARS-CoV-2 until herd immunity is reached through vaccination [5].

Researchers and many pharmaceutical companies are actively involved in the development of COVID-19 vaccines. Many different strategies and vaccine platforms have been used to design the vaccines, such as modified RNA (modRNA) in lipid nanoparticles, inactivated and attenuated whole virus vaccines, replicating and non-replicating viral vector vaccines, DNA or RNA vaccines, protein-based vaccines such as protein subunits, and virus-like particles [6,7,8,9,10,11]. Although research is ongoing, many COVID-19 vaccines have been authorised and used worldwide. These include the Oxford–AstraZeneca COVID-19, Pfizer–BioNTech COVID-19, Sputnik V COVID-19, Moderna COVID-19, Johnson & Johnson COVID-19, BBV152 and Sinovac vaccines. Despite the availability of vaccines and nationwide-scale vaccination programs in many countries, the global number of new cases can rapidly soar. Even though in recent months this could be attributed to mutations into variants of concern, as seen in the United Kingdom (variant B.1.1.7, Alpha) [12], South Africa (variant B.1.351, Beta) [13], Brazil (variant P.1, Gamma) [14] and India (variant B.1.617, Delta) [15], it could also be due to the failure of the vaccines to prevent infection and transmission, in combination with low vaccination coverage.

Despite the results of a study that involved people from 19 countries which largely point towards positive responses to COVID-19 vaccination programs, the latest information on various protective efficacies and the risks of fatal side effects of COVID-19 vaccination have changed the mind of the world population towards the usage of these vaccines [16]. This eventually leads to low vaccine coverage, and the subsequent failure to confer herd immunity, leading to continuous rises in the number of new cases. In fact, past experiences with influenza vaccination programs had confirmed this phenomenon [17].

In veterinary medicine, the issues of vaccination coverage and the rapid spread of pathogens through aerosols are common among many important viral and bacterial diseases. These include the Newcastle disease in poultry [18], rinderpest [19], foot and mouth disease [20], and haemorrhagic septicaemia in cattle and buffaloes [21]. Therefore, veterinary medicine had had substantial experience in dealing with coronaviruses of veterinary importance, which include feline infectious peritonitis (FIP), bovine enteric coronavirus infections which is closely linked to bovine respiratory disease complex (BRDC), porcine epidemic diarrhoea (PED), and infectious bronchitis (IB) that affects chicken [22]. Although most coronavirus infections in animals are efficiently controlled, some, such as PED, may be more difficult, which is largely due to frequent genetic mutations [22,23], similar to the current COVID-19 pandemic.

The delivery of vaccines via an intranasal route has long been practised, since the 17th century [24]. Now, it is widely used in both veterinary and human medicines, especially to control respiratory diseases because it stimulates mucosal immunity that prevents pathogens from colonising the respiratory tract and aids in widespread vaccination. This paper discusses the potential use of mucosal immunisation against SARS-CoV-2, based on a veterinary intranasal vaccine strategy.

## 2. Brief COVID-19 Pathogenesis

In both veterinary and human medicines, it is important to understand some basic and key events in disease development. This would help in designing efficacious treatments or preventive measures. Given that COVID-19 is a newly emerging disease, much of its detailed pathogenesis is yet to be fully understood. Nevertheless, the pathogenesis of COVID-19 is outlined into three stages [25]: (1) asymptomatic state; (2) upper airway and conducting airway responses; and (3) hypoxia, ground glass infiltrates, and progression to ARDS.

COVID-19, as with many other viral respiratory diseases of human and animals, involves the exposure and inhalation of the pathogen into the nasal cavity [25]. In this initial stage, the virus gains entry to the host and starts to establish an infection, but results in no clinical symptoms. This is similar to the enteric coronavirus infection in cats, where initial infection leads to no clinical sign or easily overlooked mild clinical signs [26]. After inhalation, the virus binds to the ciliated epithelial cells by utilising the angiotensin-converting enzyme 2 (ACE2) receptors and subsequently infects the cells and replicates [25,27]. To many veterinary scientists, this is considered a non-disease state. However, contemporary COVID-19 testing throughout the world actually detects people in this stage of infection [28], leading to overwhelming numbers of positive cases that create panic situations.

In the next stage, further propagation and migration of the virus towards the lower respiratory tract occur. These events trigger an innate immune response, and the host then starts to show clinical symptoms. A previous study revealed that the majority (80%) of patients showed only mild symptoms with total recovery within 7 to 10 days [29]. This suggests that the infection is limited to the upper conducting airways. However, as more mutations occur, as seen in the recent months or when the virus has infected people with low immunity due to concurrent health problems, a higher percentage of patients started to show severe symptoms, suggestive of invasion of the lungs. As more respiratory epithelial cells become infected, more pro-inflammatory cytokines and chemokines are produced [25].

As the infection progresses into third stage, more viral particles reach the lungs to infect the alveolar type II cells, because these cells express the ACE2 receptor and TMPRSS2 protease required for SARS-CoV-2 attachment [30]. This eventually results in more viruses, and the extensive death of alveolar type II cells. The latter incites inflammatory reactions in the lungs that make the condition worse following diffused alveolar damage, the formation of a hyaline membrane, and granulomatous inflammation with numerous alveolar macrophages. Patients that have survived ARDS may develop aberrant wound healing and significant pulmonary fibrosis [31].

Apart from pathological lesions along the respiratory tract, the ARDS in COVID-19 may also induce sepsis which is characterised by cytokine storms, especially by those proinflammatory cytokines, and microcirculation dysfunction, which eventually leads to multiple organ dysfunction syndrome [32,33]. Based on the current knowledge on the pathogenesis of COVID-19 and the general pathogenesis of respiratory diseases of human and animals, we understand the importance of halting the progression of pathogens into the lungs. Hence, strategies should be employed to improve mucosal immunity, especially in, but not limited to, the upper respiratory tract.

## 3. Intranasal Vaccine against Human and Animal Coronaviruses

Intranasal vaccination is one of the many methods of stimulating the immunity of mucosal organs such as oral, pulmonary, conjunctival, rectal, and vaginal mucosa [34]. There are many bacterial and viral infections, including COVID-19, which start from mucosal surfaces; hence, mucosal immunisation has garnered the interest of many researchers. In principle, intranasal vaccines are used to stimulate the mucosal immune system of the respiratory tract. These include mucosal lymphoid tissues (MALT) of the nasopharynx (also known as the nasopharynx-associated lymphoid tissue—NALT) and the lungs (known as bronchus-associated lymphoid tissue—BALT) [35,36]. The immune cells from these MALT function in tandem with the ciliated respiratory epithelium and goblet cells to mechanically remove the pathogens through a muco-ciliary clearance mechanism. Intranasal vaccination is known to induce innate and adaptive immunities that involve antigen-specific memory T and B cells [37]. The host B cells respond to the exposure of antigens by the production of IgA, while memory T cells are responsible for long-term protection against specific disease. Intranasal vaccination using a recombinant adenovirus-based vaccine that expresses spike proteins of Middle East respiratory syndrome coronavirus (MERS-CoV) in mice led to the presence of T cells in the respiratory airway and the lungs [38]. Intranasal boosters after an intravenous immunisation against SARS-CoV in mice also showed the presence of T cells in lungs and bronchoalveolar lavage [39]. Furthermore, many studies have pointed out that a mucosal vaccine could, in fact, induce serum IgG that further enhances vaccine efficacy [40,41,42]. Many different preparations are available that include drops, sprays, powders, gels, and solid inserts.

Many vaccines against human and animal respiratory diseases have proven to be effective when administered via the intranasal route, including the influenza vaccine for humans [43,44,45]. Some vaccines are already commercialised, whereas some are still in the clinical trial or research phases. However, intranasal vaccines against coronaviruses have not been properly studied; thus, they are not vastly available, such as the recombinant adenovirus-based MERS-CoV [38], COVID-19 [46], bovine enteric coronavirus infection, FIP, and IB [22]. To date, many vaccine developers have evaluated intranasal SARS-CoV-2 vaccines in human, which include the DelNS1-2019-nCoV-RBD-OPT1 by Beijing Wantai Biological Pharmacy and the University of Hong Kong, BBV154 by Bharat Biotech, MV-0140212 by Meissa Vaccine Inc., hAd5-S-Fusion + N-ETSD by ImmunityBio, COVI-VAC by Codagenix/Serum Institute of India, CIGB-669 by the Center for Genetic Engineering and Biotechnology, and AdCOVID by Altimmune and the University of Alabama [41,47,48,49,50,51,52]. A recent study involving rhesus macaques showed promising results, where a single dose of intranasal vaccine induced neutralising antibodies and T cell responses that prevented SARS-CoV-2 infection [53]. Major keys to the success of intranasal vaccine to prevent respiratory disease are that the disease is purely respiratory, and strong mucosal immunity develops along the respiratory tract of a vaccinated individual. The innate and adaptive mucosal immune systems serve to protect mucosal cells of the respiratory tract from invading pathogens, including SARS-CoV-2, for viral replication.

For important respiratory coronaviruses, which include SARS-CoV-2, SARS, and MERS-CoV, spike proteins are of focal interest for vaccine development. A comparative study between subcutaneous, intranasal and intramuscularly delivered whole cell-killed SARS-CoV, Spike protein, and nucleocapsid vector vaccines against SARS revealed some interesting outcomes. The whole cell-killed vaccine resulted in high serum neutralising antibodies, but not cell-mediated immune responses, which is important for controlling intracellular organisms such as viruses. Although intranasal administration of the spike protein and nucleocapsid vector produced lower serum neutralising antibodies, they significantly reduced SARS-CoV in the lungs of a murine model [54]. Similarly, a previous study that used the intranasal delivery of recombinant adenovirus-based vaccine that expressed the Spike protein of MERS-CoV showed significantly high and long-lasting S1-specific serum IgG, and the respiratory mucosal IgA [38]. This is interesting because the mucosal IgA prevents viral adhesion to the cells of respiratory tract. In fact, initial information suggests that the BBV154 intranasal vaccine against COVID-19 could stimulate a broad immune response, including that of nasal mucosa. This has high potential for controlling the COVID-19 pandemic by blocking the early establishment of viral infection and subsequent transmission of the disease [55,56]. Therefore, many speculations have been made that intranasal COVID-19 could result in better protection compared to injectable vaccines [57,58,59].

Some coronaviruses have been successfully controlled by vaccination, but others, such as PED, are difficult, largely due to frequent genetic mutations. In veterinary medicine, an intranasal vaccine was tested to investigate its effect against the bovine enteric coronavirus in feedlot cattle. It was observed that intranasal vaccination significantly reduced the incidence and the treatment for bovine respiratory disease complex, a multifactorial syndrome was partially contributed to by bovine coronavirus [60,61]. In cats, intranasal vaccination against FIP showed conflicting results [62]. Some showed great protective capability [63], whereas others showed unsatisfactory protection [64,65]. When protection was satisfactory, an intranasal vaccine was proven to be able to stimulate broad immune responses including serum IgG, serum and salivary IgA, coronavirus-neutralising antibodies, and cell-mediated immune responses [63]. Intranasal vaccination has long been applied in IB, an important respiratory coronavirus disease of chickens. Numerous vaccines are commercially available which have been shown to be protective against the infection [66,67,68]. Furthermore, adjuvanted intranasal vaccines might be able to provide better protection compared to non-adjuvanted intranasal vaccines [68].

## 4. Herd Immunity

The most important factor that prevents the outbreak of disease in a population is the level of herd immunity. Herd immunity (or population immunity for human medicine) refers to the resistance of a group or population towards a disease, because a large proportion of the members gain immunity, subsequently reducing the likelihood of an infected individual coming into contact with susceptible individuals [69,70]. In many diseases, immunity could be attained through infection or vaccination. For COVID-19, the high number of cases, increasing numbers of deaths and modelling studies which have projected hospital overloads [71] make herd immunity a priority to be achieved. Therefore, many countries aim to attain herd immunity against COVID-19 by mass vaccination so that a herd immunity threshold between 50% and 66.7% could be achieved [72,73]. Using this approach, it is important to consider vaccine efficacy in planning for mass vaccination programs.

In veterinary medicine, herd immunity is the most important aspect of vaccines and vaccination. Therefore, important aspects in vaccine development and design include: (1) the ability to confer immunity; (2) the ease of vaccine delivery; and (3) widespread vaccine coverage. An acceptable vaccine is expected to be able to provide protection to at least 75% of the herd [74]. Many vaccines that are used in animals, especially livestock animals, do not provide long-lasting protection against a specific disease. This is not a major concern because many livestock animals are reared for a short period of time. For example, a broiler chicken is reared only for about 30 days, small ruminants for 7 months, large ruminants for about a year, and certain species of fish for around 6–9 months. Exception may be seen in dairy animals, or breeder animals. For animals that are kept for a longer period of time, vaccination must be updated. For example, FMD and HS vaccines are to be administered by parenteral injection at 6-month intervals [75]. This regimen of vaccination can be expensive, laborious, and requires the training of staff, consumables, and leads to poor vaccine coverage because the majority of small and medium-scale farmers cannot afford this vaccination regimen. Therefore, many government authorities have provided assistance in vaccinating the animals, but still have not been able to achieve satisfactory herd immunity due to low vaccination coverage. In animals, another problem may lead to poor herd immunity: a rapid population turnover. The population dynamics affect herd immunity although mass vaccination has been performed [76].

In the effort to eradicate rinderpest, veterinarians across the world have had to understand the importance of mass vaccination that could stop the transmission and incidence of the disease [76,77]. Research in veterinary vaccinology focuses on developing vaccines that are easy to be delivered, which indirectly have resulted in widespread vaccine coverage. For example, vaccinations against vibriosis and streptococcosis in fish have been shown to be possible using feed-based vaccines [78,79]. Although there are many other available vaccines against these diseases that employ the injection method, the invention of feed-based vaccines that enhance mucosal immunity is seen as revolutionary and a step forward in veterinary medicine. Similarly, vaccination against rinderpest using the intranasal route was proven to be effective with comparable efficacy to a vaccine delivered by injection [80]. As for respiratory bacterial diseases in ruminants, vaccination against pneumonic mannheimiosis in small ruminants is now being performed by intranasal delivery. Several types of intranasal vaccines have been designed, including the whole cell-killed *Mannheimia* sp. to the killed recombinant bacterial vector vaccine [81]. In chickens, vaccines against economically important diseases such as Newcastle disease, IB, and infectious bursal disease (IBD) use the concept of enhancing mucosal immunity and are usually delivered by the spraying of coarse mist, or in drinking water, or by ocular drops [82,83,84].

Another approach which is used in veterinary medicine in an attempt to attain herd immunity includes the usage of live mutant pathogen or live recombinant bacteria vector vaccines. For example, a haemorrhagic septicaemia vaccine was developed by disruption of the *gdhA* gene to create a mutant *Pasteurella multocida* B:2 GDH7 strain [43,85]. This vaccine is then delivered intranasally to buffalo and cattle. This approach is considered critical, because many buffaloes and cattle in Asia and Africa, where the disease is prevalent, are reared in an extensive, free-range system for most of the year [86]. The live mutant vaccine could survive in the nasal cavity of the inoculated animals, and when these animals commingle with non-vaccinated animals, the live mutant vaccine is then transferred to the commingling animals via aerosol transmission [85]. To ensure that the mutant vaccine is self-limiting for about 2 weeks, the mutation was designed so that it disturbs the house-keeping genes for iron utilisation, which is vital for survival of the mutant in the host. This provides enough time to confer immunity to the vaccinated animals and transmission of the mutant to commingling animals. Similar outcomes may be possible using a live recombinant approach.

For ruminants, another possible approach to achieve herd immunity is through vaccination using transgenic feed materials. For example, transgenic grass that acts as a vector is capable of expressing recombinant proteins including bacterial or viral antigens [87]. However, issues such as the gene carrying capability between different generations of grass, possible contamination to the environment, and consumption by other animals may bring adverse effects to the environment.

## 5. Intranasal Vaccination as a Way Forward

In general, intranasal vaccines have immunological and non-immunological advantages over injectable vaccines. Examples of immunological advantages are the ability to stimulate both mucosal and systemic immunities, whereas the non-immunological advantages include the ease of vaccine administration, non-invasive, less discomfort, increased safety, especially when involving individuals with blood-borne diseases, and not requiring medical personnel or even making self-administration of vaccine possible, which could save time and costs for mass vaccination [88,89].

To date, a total of 102 and 185 vaccines against SARS-CoV-2 are under clinical evaluation and pre-clinical evaluation, respectively [90]. Among the vaccines that are under clinical evaluation, 76 use the intramuscular delivery route, whereas only 7 are designed for intranasal delivery. One vaccine is designed with intramuscular priming followed by an intranasal booster, while another is as an intramuscular or intranasal vaccine. These figures may suggest that the intranasal delivery route is not a popular choice in contemporary COVID-19 vaccine design. These vaccines use different platforms of non-replicating viral vectors, replicating viral vectors, live attenuated viruses, protein subunits, or inactivated viruses. Only one intranasal vaccine, DelNS1-2019-nCoV-RBD-OPT1, uses the replicating viral vector platform based on the influenza virus vector. Replicating vaccines are known to provide the most effective protection against viral infections compared to the non-replicating counterparts [91]. However, some concerns arise regarding the usage of intranasal replicating viral vector vaccines such as the potential to affect immune-compromised individuals, or prior immunity against the vector that could render the vaccine less efficacious, as well as the spread of vaccine virus in the population [91]. In veterinary medicine, replicating herpesvirus of the turkey vector vaccine is used to control poultry diseases such as IBD, ND, and avian influenza (AI), because it is safe and effective even in the presence of maternal-derived antibodies [92].

The development of human vaccines is challenging, because it should be safe, provide excellent protection, and have minimal side effects. The ease in vaccine delivery could be regarded as an added bonus. In pandemics, there is an urgent need to accelerate vaccine testing and the rollout of efficacious vaccines. Hence, it has been proposed that controlled human challenge trials should be conducted to replace Phase 3 clinical trials. This approach is disputed and deemed as an act of cutting corners [93]. Some COVID-19 vaccines were approved without Phase 3 clinical trials; therefore, the approvals were seen as premature [94], and this may lead to hesitancy [95], that later leads to poor vaccine coverage and the failure to achieve herd immunity. The recently documented severe side-effects of injectable COVID-19 vaccines are regarded as an example of vaccine hesitancy. This may be debatable; some research has suggested that side-effects such as thromboembolism do not contribute to vaccine hesitancy [96]. Some people may refuse vaccines simply because of a general lack of trust, as well as questioning the need for vaccination [95]. Even if safe and efficacious vaccines are designed in the near future, this stigma may persist. Although vaccine hesitancy and refusal have been reported in veterinary practices [97], its effect may not be as costly compared to the current pandemic situation or other human diseases [98].

COVID-19 is a respiratory disease; therefore, similar strategies that are used in controlling animal respiratory diseases could be employed, with the main aim of achieving widespread vaccination coverage. For example, using a coarse mist approach, or intranasal vaccination with live recombinant vaccine, or live mutant. Although this is actually possible, bioethical issues are of concern, particularly the effects on immune-compromised individuals and the spread of a vector in the population. The administration of a replicating attenuated or mutant vaccine to a human and hope that the vaccine is transmitted to other individuals is ideal to attain quick and widespread vaccine coverage that ensures herd immunity. Theoretically, this approach is highly beneficial to increase vaccine coverage, especially in less wealthy countries. This aligns well with the COVAX plan devised by the World Health Organization and the general goal to achieve health equity [99]. However, a commingling individual may lose the freedom to choose their vaccination status. Issues pertaining to making it mandatory for vaccination against COVID-19 or other diseases have been discussed [100,101,102], with the general inclination towards maintaining vaccination a choice.

## 6. Conclusions

Veterinarians have their fair share of experiences in dealing with coronavirus infections, fatal respiratory diseases, and the eradication of diseases. Although the approach of vaccination is slightly different compared to human medicine, the main aim is similar; to reduce the incidence of disease largely by achieving herd immunity. Intranasal vaccination is deemed as a very useful delivery method, especially to control important respiratory diseases in animals and human as well as to attain widespread vaccine coverage. However, depending on the vaccine platform, intranasal vaccination in humans should be approached carefully to avoid issues pertaining to bioethics, vaccine hesitancy, and vaccine refusal.

## Data Availability

Not applicable.
